# Dynamic Acquisition of [^99m^Tc]Tc-Pyrophosphate SPECT/CT Images in Transthyretin Cardiac Amyloidosis: A Pilot Study

**DOI:** 10.2967/jnumed.125.270405

**Published:** 2026-02

**Authors:** Benjamin Auer, Alyssa De Moraes, Ardel J. Romero Pabon, Olivier F. Clerc, Sudhir Bhimaniya, Marie Foley Kijewski, Annu Kurian, Shilpa Vijayakumar, Sarah A.M. Cuddy, Marcelo F. Di Carli, Sharmila Dorbala

**Affiliations:** Division of Nuclear Medicine and Molecular Imaging, Department of Radiology, Brigham and Women’s Hospital, Boston, Massachusetts

**Keywords:** transthyretin cardiac amyloidosis, [^99m^Tc]Tc-pyrophosphate, dynamic SPECT imaging, quantitative SPECT

## Abstract

The objectives of this study were to determine the timing of peak [^99m^Tc]Tc-pyrophosphate uptake in the myocardium, blood pool, and bone and to explore the feasibility and advantage of early imaging compared with the standard late imaging in participants with and without transthyretin amyloid cardiomyopathy (ATTR-CM). **Methods:** Dynamic [^99m^Tc]Tc-pyrophosphate SPECT/CT data were acquired at 0–5 min and 10–65 min, with additional 15-min static scans at 90 and 150 min using a full-ring cadmium zinc telluride scanner. Image analysis included visual assessment and established quantitative metrics that require calibrated SPECT images, such as SUV_mean_ and SUV_max_ and percentage injected dose per milliliter, along with relative uptake ratios (myocardium to bone and myocardium to blood pool) that do not require calibration. ATTR-CM and non–ATTR-CM cohorts were compared. **Results:** Our study included 19 participants: 8 with ATTR-CM (median age of 80 y with an interquartile range of 9.3 y) and 11 (57.9%) without ATTR-CM. In ATTR-CM, SUV_mean_ was significantly higher in the myocardium than in the blood pool at 10 min (3.86 ± 0.77 vs. 3.08 ± 0.58, *P* = 0.0055). Myocardial SUV_mean_ remained elevated over time, with a statistically significant decline from 3.86 ± 0.77 at 10 min to 2.88 ± 0.57 at 150 min (*P* = 0.0025). In patients without ATTR-CM, myocardial SUV_mean_ remain relatively stable across all time points (1.24 ± 0.41 at 10 min to 0.94 ± 0.21 at 150 min, *P* = not statistically significant). In both groups, bone SUV_mean_ peaked at 90 min, and the blood pool showed the highest SUV at 10 min, followed by a decrease through 150 min. Percentage injected dose per milliliter clearly separated the ATTR-CM from the non–ATTR-CM groups at all time points. **Conclusion:** In participants with ATTR-CM, myocardial uptake peaked by 10 min after injection and remained stable, with minimal washout, through 150 min; non–ATTR-CM participants showed consistently lower myocardial uptake than blood pool uptake at all time points. These findings suggest that early imaging can perform as well as late imaging for diagnosis of ATTR-CM, supporting the potential of early imaging to streamline patient care.

## Visual Abstract

**Figure:**
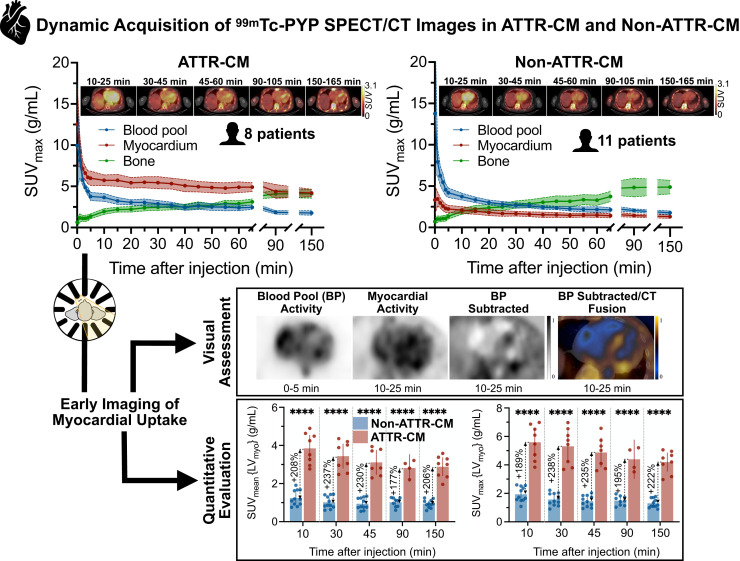

Cardiac SPECT with [^99m^Tc]Tc-pyrophosphate (^99m^Tc-PYP) is an established noninvasive method for diagnosing transthyretin amyloid cardiomyopathy (ATTR-CM). Visual image interpretation comparing myocardial to bone uptake using the Perugini grading scale is the current clinical standard. Perugini grades 2 and 3 (myocardial uptake equal to bone uptake and greater than bone uptake, respectively) are considered diagnostic for ATTR-CM, with a specificity of nearly 100%, but this approach is only about 72% sensitive (1). Current guidelines recommend imaging 2–3 h after injection of ^99m^Tc-PYP (2,3). This prolonged uptake period allows for clearance of radiotracer from the blood pool, improving the myocardium–to–blood pool contrast and reducing the likelihood of false positives. This delay also allows for radiotracer accumulation and stabilization in bones, essential for Perugini visual grading. Importantly, although the goal is to image myocardial ^99m^Tc-PYP uptake to diagnose ATTR-CM, the postinjection scan delay is not chosen to maximize myocardial radiotracer uptake or minimize tracer washout. It is thus possible that the 2- to 3-h delay reduces test sensitivity, especially since the precise timing of peak myocardial uptake in ATTR-CM remains uncertain. Quantitative ^99m^Tc-PYP SPECT/CT eliminates the need for visual comparison to bone uptake and optimizes acquisition timing to match peak myocardial activity.

Emerging evidence from 2-dimensional planar imaging suggests that myocardial uptake in ATTR-CM occurs earlier than 2–3 h after injection (4–7). However, to our knowledge, no studies to date have evaluated myocardial uptake over time using ^99m^Tc-PYP, nor have any studies used SPECT or SPECT/CT imaging. Our study addressed this gap by analyzing the uptake and washout of ^99m^Tc-PYP in the myocardium, blood pool, and bone over 2.5 h in both ATTR-CM and non–ATTR-CM participants. We used dynamic imaging on a full-ring SPECT/CT scanner equipped with cadmium zinc telluride (CZT) semiconductor detectors to quantify ^99m^Tc-PYP uptake over time.

The objectives of this study were to determine the uptake of ^99m^Tc-PYP over time in the myocardium, blood pool, and bone in participants with and without ATTR-CM. We also explored the feasibility and advantage of imaging soon after injection of ^99m^Tc-PYP compared with imaging at 2.5 h using visual assessment, absolute calibration-dependent uptake metrics, and calibration-independent relative ratios.

## MATERIALS AND METHODS

### Study Population

This pilot study included 19 participants referred clinically or for research ^99m^Tc-PYP SPECT/CT imaging at Brigham and Women’s Hospital between April 2023 and January 2025. The study was approved by our Institutional Review Board, and all subjects provided written informed consent. Demographics, past medical history, and clinical red flags for amyloidosis were identified through review of electronic medical records. All participants tested negative for plasma cell dyscrasia, as determined by serum free-light-chain assay and serum and urine immunofixation electrophoresis. Transthyretin genetic testing was performed on all participants with positive ^99m^Tc-PYP scan results.

### Dynamic SPECT/CT Acquisition Protocol

**FIGURE 1.**
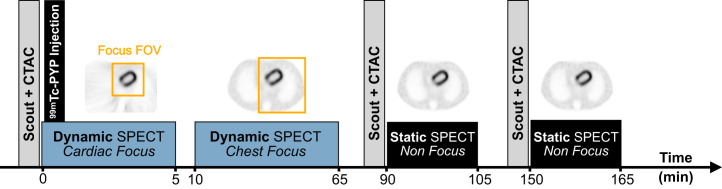
Study protocol. CTAC = attenuation-corrected CT; FOV = field of view.

Given the novelty of the dynamic SPECT/CT acquisition protocol used in this work, we have provided additional details on calibration, acquisition, and reconstruction in the supplemental materials (available at http://jnm.snmjournals.org). Participants were injected intravenously with 887.9 ± 84.8 MBq (24.0 ± 2.3 mCi) of ^99m^Tc-PYP and were scanned on a first-generation full-ring CZT SPECT/CT system (Veriton 200 series; Spectrum Dynamics Medical) (Supplemental Sections A, B, C, and D). Dynamic SPECT/CT acquisition was initiated a few seconds before radiotracer injection with the patient’s arms positioned down. As shown in Figure 1, image acquisition consisted of a 5-min dynamic scan focused on the heart, followed by a 55-min dynamic acquisition covering the heart, ribs, and spine. Additional 15-min nonfocused static chest SPECT/CT acquisitions were performed at 1.5 and 2.5 h after injection, with an average delay of 89.7 ± 16.7 min and 153.4 ± 6.7 min, respectively. These static SPECT acquisitions involved a limited number of gantry rotations, typically 3 or 4 depending on the participant body contour, to improve overall angular sampling and, thus, image quality. Images were acquired using a ^99m^Tc energy window of ±10% (125–155 keV) centered at 140 keV. Veriton software version 2.5 was used for image acquisition and reconstruction. The full-ring CZT SPECT/CT system was calibrated for absolute quantitation by acquisition of images of a uniform cylindric phantom filled with a known ^99m^Tc activity using the nonfocused static imaging protocol (Supplemental Section E).

### Image Reconstruction

Dynamic 4-dimensional SPECT acquisitions with a cardiac or chest focus were reconstructed using ordered-subset expectation maximization with 3 iterations, 16 subsets, a voxel size of 4.92 mm (128 × 128 matrix), and an intrareconstruction convolution filter (frequency of 0.125, order of 2). The reconstruction algorithm incorporates resolution recovery using analytic point-spread function modeling, CT-based attenuation correction, and spline fitting between time frames. Nonfocused static SPECT acquisitions were reconstructed with similar parameters, except that an enhanced proprietary point-spread function modeling approach including collimator and object scatter correction was used (Supplemental Section D).

### Expert Image Interpretation

^99m^Tc-PYP static SPECT/CT images acquired 2.5 h after injection were visually evaluated by a single expert physician with over 20 y of experience. Myocardial ^99m^Tc-PYP uptake was graded according to the Perugini scale. A visual score of 2 or greater was considered diagnostic for ATTR-CM. Fused SPECT/CT images were used to help visually distinguish myocardial from blood pool uptake.

### SPECT/CT Image Segmentation

**FIGURE 2.**
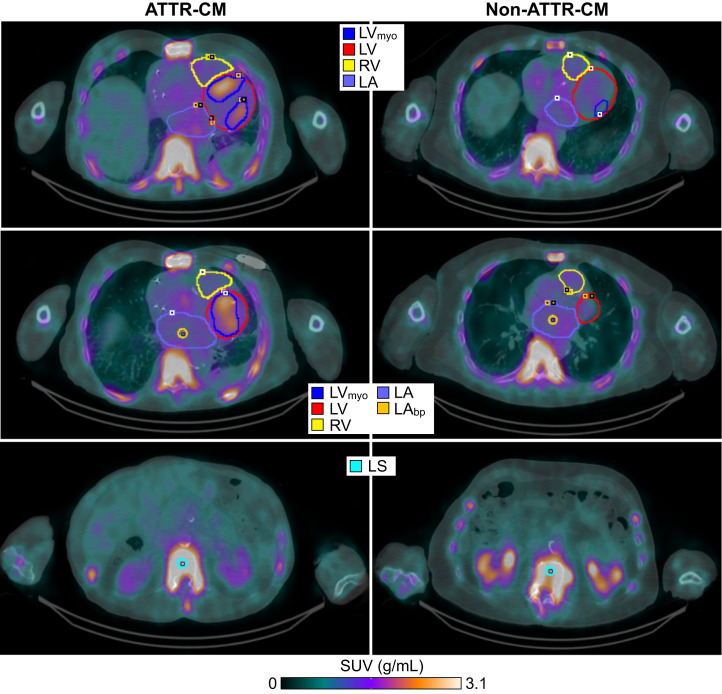
Regions of interests for ATTR-CM and non–ATTR-CM. (Top) LV_myo_, left ventricle (LV), right ventricle (RV), and left atrium (LA) VOIs. (Middle) LV_myo_, LV, RV, LA, and LA_bp_. (Bottom) Bone contour (LS) drawn in central cancellous vertebral region along spine.

Multiple volumes of interest (VOIs) were delineated on 2.5-h-postinjection clinical SPECT images using coregistered CT images for anatomic guidance on PMOD Fusion software (PMOD Technologies LLC) as described in Supplemental Section F. VOIs were placed in the left ventricular myocardium (LV_myo_), left atrial blood pool (LA_bp_), and lower thoracic spine (LS), as shown in Figure 2 for both a positive and a negative case. All VOIs were subsequently imported and minimally adjusted on the dynamic and 1.5-h static images to ensure accurate alignment without altering VOI dimensions significantly.

### Image Analysis

Quantitative analysis focused on 3 primary VOIs (LA_bp_, LV_myo_, and LS) relevant to the diagnosis of ATTR-CM. Temporal variation of ^99m^Tc-PYP uptake was evaluated using SUV_mean_, SUV_max_, and myocardial percentage injected dose per milliliter (%ID/mL), along with semiquantitative SUV_mean_ ratios of myocardium to bone (LV_myo_/LS) and myocardium to blood pool (LV_myo_/LA_bp_). SUV_mean_ and SUV_max_, expressed in g/mL, were defined as the average and maximum activity concentrations within a given VOI, normalized to the injected activity per unit of patient body weight. The activity concentrations used for SUV calculation were decay-corrected to the time at which the injected activity was measured. The injected activity was corrected for residual activity measured after injection. The %ID/mL was calculated as the SUV_mean_ in LV_myo_ divided by the body weight, providing a body-weight–independent quantitative metric. Additional details can be found in Supplemental Section G. For comparative analysis over time between dynamic and static images, dynamic image frames were averaged over three 15-min intervals: 10–25 min, 30–45 min, and 45–60 min. These synthetic static images were then compared qualitatively and quantitatively, using the same metrics, with the 15-min static images acquired at 1.5 and 2.5 h after injection. All quantitative analyses were supported by visual review of time-series images. Two experienced readers in ATTR-CM imaging independently reviewed the early-time-point images masked to the time of acquisition, clinical information, and late image findings. Visual assessment was based on a blood pool grading scale, where myocardial uptake equal to or greater than blood pool uptake was considered positive and myocardial uptake less than blood pool uptake was considered negative. The clinical scan at 2.5 h after injection, interpreted using the Perugini scale, served as the ground truth. Diagnostic performance in terms of sensitivity, specificity, and accuracy was calculated for each reader.

### Statistical Analysis

Categoric variables were summarized as counts and percentages and compared using the Fisher exact test or the Pearson χ^2^ test, as appropriate. Continuous variables were tested for normality using the Shapiro–Wilk test. Normally distributed variables were compared using unpaired Student *t* tests, and nonnormally distributed variables were compared using the Wilcoxon rank-sum test. Data are reported as mean ± SD for normally distributed variables and as median with interquartile range for nonnormally distributed variables. Repeated-measures ANOVA with Tukey post hoc and Geisser–Greenhouse correction was used to compare quantitative ^99m^Tc-PYP metrics across multiple periods. Two-tailed unpaired *t* tests were used to compare quantitative metrics between ATTR-CM and non–ATTR-CM groups at different periods. Interobserver agreement at early time points was assessed using the Cohen κ-statistic. All statistical analyses were performed using GraphPad Prism version 10.4.2, with a *P* value of less than 0.05 considered statistically significant.

## RESULTS

### Study Cohort

Baseline characteristics of the study cohort are summarized in Table 1. In total, 19 participants were included in this study, 8 with a diagnosis of wild-type ATTR-CM (42.1%) and 11 (57.9%) without ATTR-CM. Among the ATTR-CM cases, 3 participants (37.5%) had initiated treatment with tafamidis before imaging, with a short average duration of 1.7 ± 1.1 wk at the time of the scan. Although no significant difference in body surface area or weight was observed between groups, a modest but statistically significant difference in body mass index was noted.

**TABLE 1.** Baseline Characteristics

Characteristic	Non–ATTR-CM (*n* = 11)	ATTR-CM (*n* = 8)	*P*
Demographic			
Median age (y)	66.0 (IQR, 18.5)	80.0 (IQR, 9.3)	0.07
Male	7 (63.6)	8 (100)	0.10
Body surface area (m^2^)	2.00 ± 0.22	1.92 ± 0.24	0.47
Body weight (kg)	89.0 ± 15.8	79.1 ± 17.2	0.22
Body mass index (kg/m^2^)	30.9 ± 5.6	26.4 ± 3.4	0.04
Medical history			
Hypertension	7 (63.6)	8 (100)	0.10
Hyperlipidemia	9 (81.8)	8 (100)	0.48
Heart failure	8 (72.7)	8 (100)	0.23
Coronary artery disease	5 (45.5)	3 (37.5)	0.99
Diabetes	1 (9.1)	1 (12.5)	0.99
Smoking	0 (0)	0 (0)	0.99
Pedal edema	4 (36.4)	3 (37.5)	0.99
Stroke	1 (9.1)	1 (12.5)	0.99
Musculoskeletal^†^	0 (0)	5 (62.5)	0.01
New York Heart Association class ≥ II	6 (60.0)	6 (75.0)	0.64
Amyloid red flags			
Bilateral carpal tunnel syndrome	5 (45.5)	2 (25.0)	0.63
Spinal stenosis	3 (27.3)	2 (25.0)	0.99
Peripheral neuropathy	6 (54.5)	7 (87.5)	0.18
Aortic stenosis (any degree)	0 (0)	0 (0)	0.99

† Musculoskeletal includes carpal tunnel syndrome, biceps tendon rupture, lumbar spinal stenosis.

IQR = interquartile range.

Qualitative data are number and percentage; continuous data are mean ± SD, except for age.

### Early Temporal Changes in Quantitative Metrics

**FIGURE 3.**
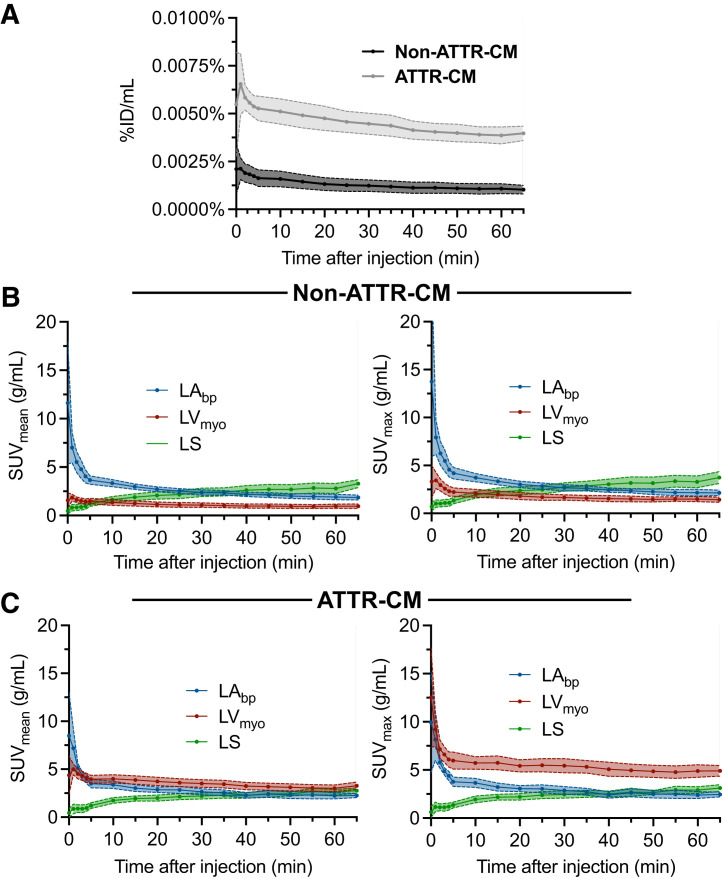
(A) Myocardial %ID/mL for non–ATTR-CM and ATTR-CM groups for first 65 min after injection. (B and C) SUV_mean_ (B) and SUV_max_ (C) for non–ATTR-CM and ATTR-CM groups in LA_bp_, LV_myo_, and LS for first 65 min after injection. Data are shown as average ± 95% CI.

Analysis of the dynamic images revealed distinct uptake patterns for the ATTR-CM and non–ATTR-CM groups within the first 65 min after injection (Fig. 3). There was clear separation of study groups by %ID/mL over time (Fig. 3A). In ATTR-CM, %ID/mL peaked early (∼0.007% at 2.5 min) and gradually decreased to about 0.004% by 65 min, whereas in non–ATTR-CM participants, it reached a maximum of about 0.002% at 1 min and declined to about 0.001% over the same period. Overall, for each group, SUV_mean_ and SUV_max_ showed similar trends across myocardium, blood pool, and bone regions (Figs. 3B and 3C). In the non–ATTR-CM cohort (Fig. 3B), blood pool SUVs were consistently higher than SUVs in the myocardium throughout the 65 min, despite a gradual decline over time. Myocardial uptake peaked rapidly after injection (within the first 10 min) and remained relatively stable after that. Blood pool uptake similarly peaked early but declined slowly. Bone uptake increased more gradually, surpassing myocardial uptake after approximately 10 min and blood pool concentration after around 40 min. In the ATTR-CM group (Fig. 3C), blood pool SUVs were initially higher than myocardial SUVs during the first few minutes after injection but were below myocardial SUVs by around 10 min. Thereafter, myocardial SUVs remained consistently higher than SUVs in the blood pool, with minimal washout. The separation between myocardium and blood pool time–activity curves was, however, more pronounced in SUV_max_ than in SUV_mean_. Bone uptake showed a slow accumulation pattern similar to that in the non–ATTR-CM group, surpassing blood pool SUVs at around 40 min but remaining lower than myocardial SUVs through 65 min.

### Variation of Quantitative Metrics over 2.5 Hours

To make our results more clinically applicable to static images, we analyzed the 15-min synthetic static images reconstructed from the dynamic acquisitions at 10–25 min, 30–45 min, and 45–60 min after injection, alongside 90-min (1.5-h) and 150-min (2.5-h) static acquisitions. These results revealed that the distinct temporal patterns of SUV_mean_ and SUV_max_ in the first 65 min persisted for 150 min between non–ATTR-CM and ATTR-CM participants (Supplemental Figs. 2 and 4).

**FIGURE 4.**
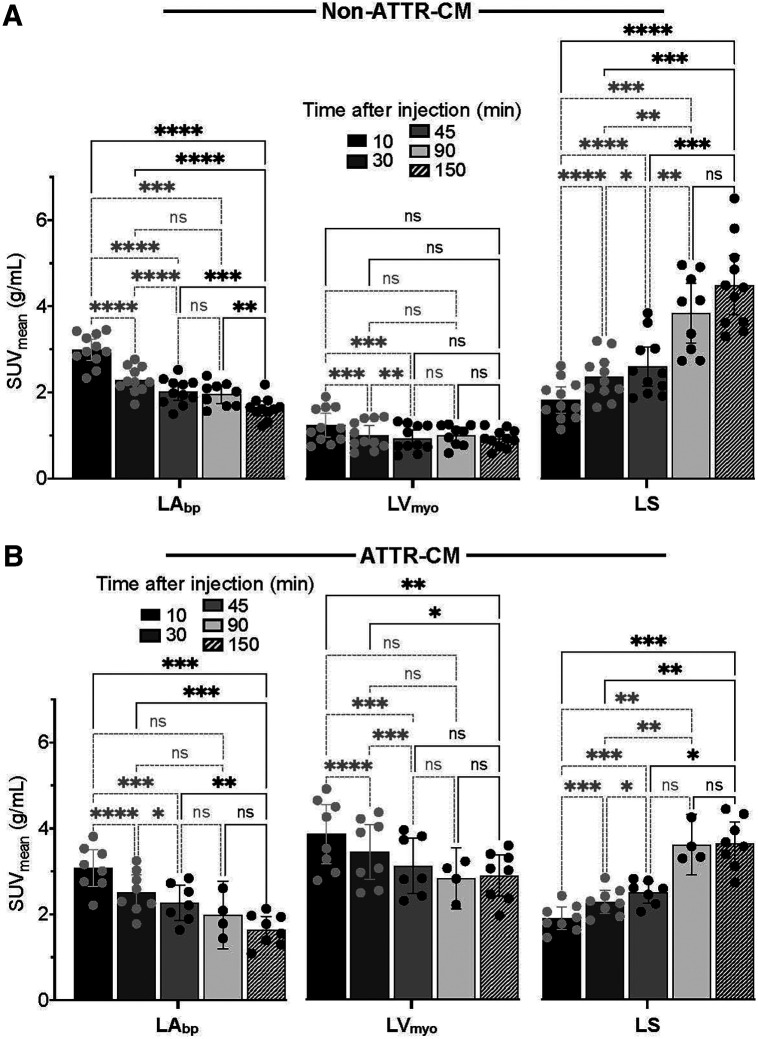
SUV_mean_ for non–ATTR-CM (A) and ATTR-CM groups (B) for 15-min static images (generated by time-averaging dynamic frames) at 10, 30, and 45 min after injection and for 15-min static images acquired at 90 and 150 min. Data are presented as average ± 95% CI, with individual values shown as dots. *P ≤ 0.05. **P ≤ 0.01. ***P ≤ 0.001. ****P ≤ 0.0001. ns = not significant.

In the non–ATTR-CM group, blood pool SUVs peaked at 10 min (SUV_mean_, 2.99 ± 0.51), followed by a significant decline by 30 min (1.64 ± 0.26, *P* < 0.0001) and a more gradual decrease through 150 min (1.64 ± 0.26, *P* < 0.0001) (Fig. 4). Myocardial SUV_mean_ remained relatively stable across all time points, decreasing only slightly from 1.24 ± 0.41 at 10 min to 0.94 ± 0.21 at 150 min (*P* > 0.05), indicating minimal washout. Bone SUV_mean_ increased steadily, peaking at 90 min (from 1.83 ± 0.55 at 10 min to 4.40 ± 1.14 at 90 min, *P* = 0.0003). In the ATTR-CM group, myocardial uptake remained elevated and relatively stable across time, with a statistically significant decline from 10 min to 150 min (3.86 ± 0.77 to 2.88 ± 0.57 for SUV_mean_, *P* = 0.0025). Importantly, even at 10 min, myocardial SUV_mean_ was already significantly higher than blood pool SUV_mean_ (3.86 ± 0.77 vs. 3.08 ± 0.58, *P* = 0.0055). Bone uptake followed a gradual accumulation as in the negative cohort, peaking at 90 min, but remained consistently lower than myocardial activity. A similar trend was observed with SUV_max_ in the myocardium, blood pool, and bone (Supplemental Fig. 2).

**FIGURE 5.**
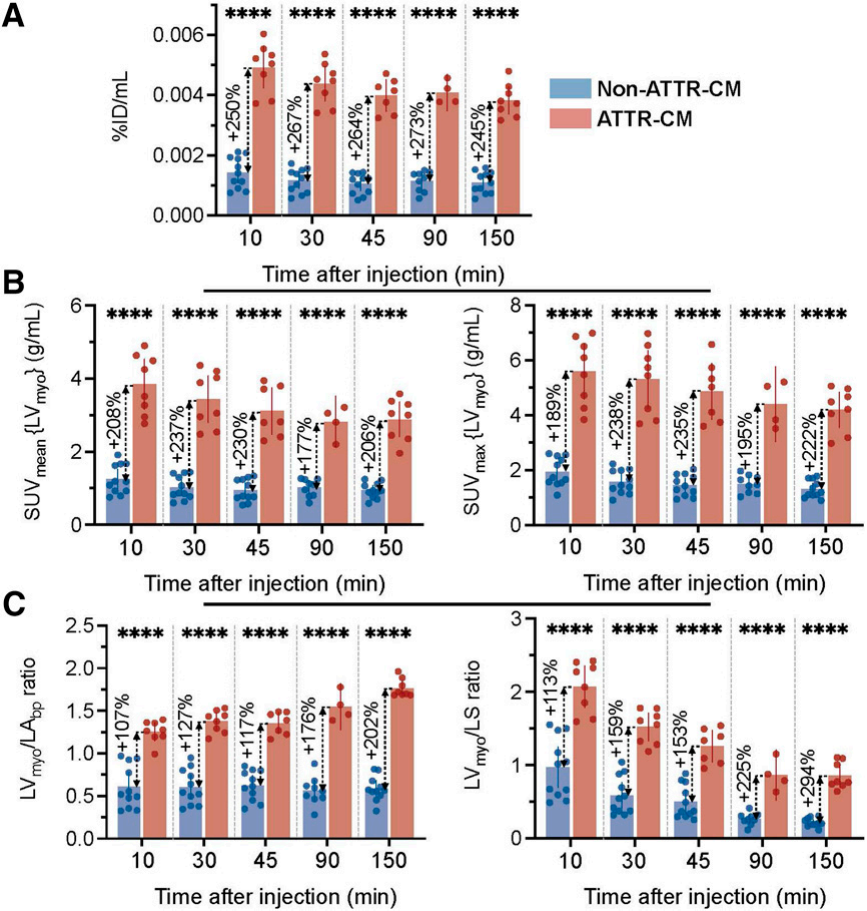
Comparison of absolute calibration-dependent and relative myocardial quantitative metrics between non–ATTR-CM and ATTR-CM groups for different periods. (A) Myocardial %ID/mL. (B) SUV_mean_ (left) and SUV_max_ (right) in LV_myo_. (C) Semiquantitative SUV_mean_ ratios of myocardium to blood pool (LV_myo_/LA_bp_, left) and myocardium to bone (LV_myo_/LA_bp_, right). Data are presented as average ± 95% CI, with individual values shown as dots. *P ≤ 0.05. **P ≤ 0.01. ***P ≤ 0.001. ****P ≤ 0.0001. ns = not significant.

Results between negative and positive groups evaluated on a per-participant basis are shown using a bar graph with dot plots (Supplemental Figs. 5A–5C). After 30 min, all measures (%ID/mL, SUV_mean,_ SUV_max,_ LV_myo_/LA_bp_ ratio, and LV_myo_/LS ratio) distinguished ATTR-CM from non–ATTR-CM without overlap. But at the early time point of 10 min, only the absolute calibration-dependent uptake myocardial metrics of %ID/mL (Fig. 5A), SUV_mean_ (Fig. 5B), and SUV_max_ (Fig. 5B) showed perfect separation of ATTR-CM and non–ATTR-CM groups. By contrast, relative target-to-background ratios (LV_myo_/LV_bp_ ratio, LV_myo_/LS ratio; Fig. 5C) showed overlap. Although these relative ratios offered lower diagnostic separation than absolute calibration-dependent metrics, they had the advantage of not requiring absolute calibration and on average were consistently higher in ATTR-CM participants. Myocardial %ID/mL is a robust quantitative metric for consistent and stable group separation across all periods. ATTR-CM participants showed a %ID/mL of more than 0.003% at all time points, whereas values for non–ATTR-CM participants remained below that threshold, with minimal variability.

### Feasibility of Early Imaging

**FIGURE 6.**
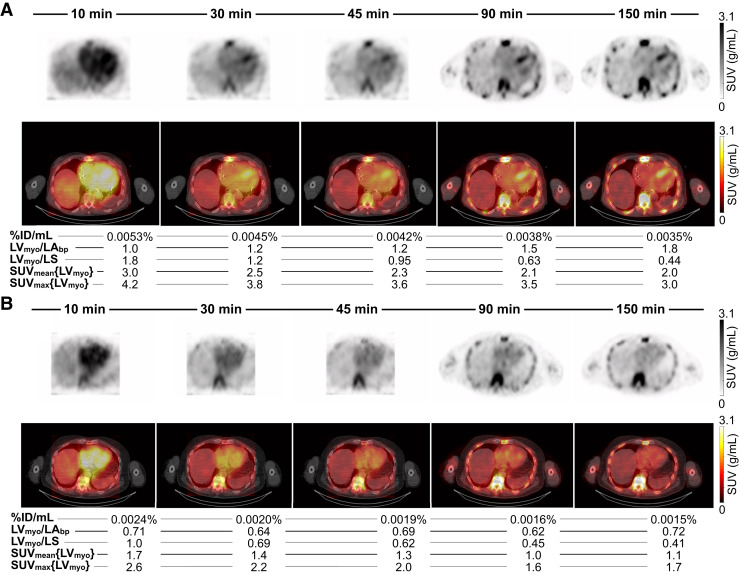
ATTR-CM (A) and non–ATTR-CM (B) examples showing 15-min images and associated average quantitative metrics at 10, 30, 45, 90, and 150 min after injection. (A) ATTR-CM example in 83-y-old man on tafamidis therapy for 1.1 wk. Dynamic acquisition was performed after injection of 884 MBq (23.9 mCi) of ^99m^Tc-PYP. Myocardial uptake can be seen as early as 10 min after injection. Quantitative metrics (SUV_mean_, SUV_max_, %ID/mL, and LV_myo_/LS ratio) peaked at early time points, whereas LV_myo_/LA_bp_ ratio increased over time because of progressive blood pool washout. Bone uptake gradually increased over time, limiting applicability of Perugini scale for early imaging (<90 min). (B) Non–ATTR-CM example in 85-y-old man injected with 888 MBq (24.0 mCi) of ^99m^Tc-PYP. Myocardial uptake remained consistently similar to or lower than blood pool activity across all time points, with no evidence of progressive myocardial retention.

Visual interpretation of images supported the feasibility of early imaging (Fig. 6A, ATTR-CM; Fig. 6B, non–ATTR-CM). In the ATTR-CM participant, myocardial uptake was already present at 10 min, although contrast was suboptimal because of high blood pool activity. By 30 min, myocardium–to–blood pool contrast improved as activity washed out from the blood pool, whereas myocardial uptake remained stable, leading to a more interpretable image. In contrast, in the non–ATTR-CM participant, the myocardial concentration was less than the blood pool concentration at 10 min and subsequently was not distinguishable from the blood pool concentration at any time point, with blood pool uptake dominating at early times and bone uptake increasing progressively over time. These participants showed no myocardial accumulation at later times, indicating that delayed imaging may be unnecessary in such cases. In this pilot study, diagnostic performance in terms of sensitivity, specificity, and accuracy was perfect (100%) at all early time points for both readers (Supplemental Fig. 3). Interobserver agreement was also perfect, with a κ-value of 1.00 at 10, 30, and 45 min after injection (Supplemental Tables 2–4).

## DISCUSSION

In this pilot study, we used quantitative dynamic ^99m^Tc-PYP imaging, applied for the first time in the context of cardiac amyloidosis (to our knowledge), using a full-ring CZT SPECT/CT system, to evaluate temporal variations in ^99m^Tc-PYP concentration in participants with and without ATTR-CM. Our study revealed several novel and clinically relevant findings. First, myocardial uptake in ATTR-CM participants was distinguishable from blood pool activity as early as 10 min after injection, quantitatively more than visually, with the SUV_mean_ and SUV_max_ of the myocardium being consistently higher than those of the blood pool, but with modest statistically significant washout, and remaining stable through 2.5 h after injection. These findings are aligned with the diagnostic accuracy reported for current clinical protocols with delayed imaging at 60 and 150 min. Second, blood pool activity peaked early and progressively cleared in both groups, improving myocardium–to–blood pool contrast over time, with early images acquired between 10 and 45 min already visually interpretable. Third, bone uptake increased gradually, peaking at around 90 min after injection; therefore, visual grading using the Perugini scale is unsuitable at early time points when bone uptake has not yet maximized. Fourth, the myocardial %ID/mL was consistently lower in the negative group than in the ATTR-CM group from the earliest time points onward, supporting the potential of early quantitative imaging to reliably distinguish positive from negative cases. Notably, in this pilot cohort, delayed imaging could have been avoided in those with a %ID/mL of less than 0.003% at the 10- to 30-min time point; this threshold needs further validation.

Using late imaging findings as the reference standard, visual interpretation of early images based on a myocardium–to–blood pool grading scale demonstrated perfect interobserver agreement and diagnostic performance. However, even for expert readers, visual interpretation of ^99m^Tc-PYP images at early time points can remain difficult because of high blood pool uptake and the inapplicability of the Perugini grading scale. To facilitate visual interpretation of early myocardial uptake, we propose a simple and novel approach based on subtracting blood pool activity, with the first 5 min of dynamic data being averaged to generate blood pool images (Supplemental Section H; Fig. 4). This approach might aid in distinguishing myocardial from blood pool activity for the ATTR-CM group.

Early imaging within the first hour after injection has been explored with other bone-avid radiotracers, such as [^99m^Tc]Tc-3,3-diphosphono-1,2-propanodicarboxylic acid (4,6) and [^99m^Tc]Tc-hydroxymethylene diphosphonate (5,7); however, such studies relied on planar imaging with associated semiquantitative metrics. Santarelli et al. demonstrated the feasibility of detecting ATTR-CM using [^99m^Tc]Tc-hydroxymethylene diphosphonate time–activity curves and retention indices as early as 6 min after injection (7), and Minutoli et al. reported similar diagnostic performance using early-phase whole-body [^99m^Tc]Tc-3,3-diphosphono-1,2-propanodicarboxylic acid planar imaging (6). In contrast, early imaging with ^99m^Tc-PYP, as well as temporal changes in myocardial uptake, remain largely underinvestigated, with no studies, to our knowledge, of imaging before the 1-h-postinjection mark and none assessing absolute myocardial quantitation derived from calibrated SPECT/CT imaging.

Our study addressed this gap using a full-ring CZT SPECT/CT system. The use of this advanced imaging technology has previously been limited to technical notes, renal imaging of a single pig (8), a single case report of a 3-phase bone scan (9), and a single case report of a myocardial perfusion study (10). In our study, in addition to the human data on 19 participants, we performed a uniform phantom scan acquired with both dynamic and static imaging protocols and confirmed equivalent quantitative accuracy between dynamic and static acquisition modes (Supplemental Section E).

Relying solely on visual assessment to reliably diagnose ATTR-CM at early imaging time points remains inherently limited. Our results imply that myocardial uptake quantitation using myocardial SUV_mean_, SUV_max_, and %ID/mL metrics represents a more objective and robust alternative. ATTR-CM and non–ATTR-CM groups could be separated both visually and quantitatively, especially using myocardial %ID/mL, at all time points, even at 10 min after injection (Supplemental Table 1), because myocardial uptake exceeds blood pool uptake from early on. Additionally, myocardial and blood pool uptake stabilized after approximately 10 min after injection (Fig. 3), suggesting that early static imaging initiated at or after this time point may be feasible on conventional dual-head systems without introducing significant artifacts from rapid radiotracer redistribution, though further validation is needed. Fully automated diagnostic tools based on artificial intelligence–driven segmentation of CT attenuation images are now available or under development (11,12). These tools may facilitate broader clinical adoption of absolute myocardial quantification and support the practical implementation of quantitative early imaging in routine practice. In this context, CT attenuation images play an essential role that extends beyond myocardial segmentation for quantitation; they also enhance visual interpretation by aiding in the differentiation of myocardial uptake from blood pool activity.

Our study had several limitations. First, the cohort size was limited, primarily because of the demands of our extended imaging protocol and the older age of the participants, but statistically significant differences were observed, supporting the adequacy of our sample size. Second, %ID/mL has been shown to be dependent on body size, age, and sex. Since we observed group differences in body mass index and no consensus exists on the optimal normalization method, our results should be interpreted with caution. Third, a subset of ATTR-CM participants was receiving tafamidis therapy for a short duration (<3 wk) at the time of imaging, which may have minimally underestimated myocardial uptake patterns. Fourth, image quality for the dynamic and synthetic static images was inferior to that for true static images, because of reduced angular sampling (i.e., a single orbit without gantry rotation) and the lack of scatter correction and enhanced point-spread function modeling, which were not available for the dynamic reconstructions. Lastly, myocardial segmentation was derived from late images using a semiautomated blood pool–based thresholding approach. Independent contouring of early images using the late imaging threshold was not feasible because of higher blood pool activity; however, an appropriate threshold specific to early imaging must be established in future investigations.

Despite these limitations, our findings clearly suggest that early imaging, particularly between 10 and 45 min after injection, is feasible to confidently differentiate ATTR-CM from non–ATTR-CM cohorts.

## CONCLUSION

Applying innovative technology in quantitative dynamic SPECT/CT imaging using a full-ring CZT SPECT/CT system in humans, we investigated the time course of ^99m^Tc-PYP uptake in participants with and without ATTR-CM. In ATTR-CM participants, myocardial uptake exceeded blood pool activity as early as 10 min after injection and remained stable, with minimal washout, through 2.5 h, as reflected by quantitative metrics including SUV_mean_, SUV_max_, and %ID/mL. In contrast, non–ATTR-CM participants showed consistently lower myocardial uptake than blood pool activity from the earliest time points. These findings suggest that early imaging performs well, supporting its promising potential to streamline patient care. Future research should aim to validate these results in larger cohorts by evaluating the blood pool subtraction approach introduced, determining the myocardial isocontour threshold adapted to early imaging, and assessing the diagnostic performance of the quantitative myocardial metrics.

## DISCLOSURE

Benjamin Auer received travel reimbursement for speaking engagements from Spectrum Dynamics Medical, as well as in-kind research support from GE HealthCare. Olivier Clerc was supported by NIH grant K99HL175107 and a research fellowship from the International Society of Amyloidosis and Pfizer. Sarah Cuddy received grant support from NIH (1K23HL166686-01 and AHA 23CDA857664) and personal (speaking/consulting) fees from Pfizer, Bridgebio, Ionis, AstraZeneca, Alexion, Novo-Nordisk, Life Molecular Imaging, and Attralus. Marcelo Di Carli received research funding from Gilead Sciences, Xylocor, Sun Pharma, Intellia Therapeutics, Alnylam; in-kind research support from Amgen; and consulting fees from MedTrace, Valo Health, IBA, and Sanofi. Sharmila Dorbala received consulting fees from Pfizer, GE HealthCare, and MedTrace; research funds from Attralus, GE HealthCare, Pfizer, Siemens, BridgeBio, and AstraZeneca; and NIH research support (K24 HL157648, R01HL159987, and R01HL150342). No other potential conflict of interest relevant to this article was reported.

## Notes

KEY POINTS **QUESTION:** What is the time course of maximal myocardial uptake of ^99m^Tc-PYP in ATTR-CM, and is early imaging at this time a reliable alternative to delayed imaging for accurately quantifying myocardial uptake? **PERTINENT FINDINGS:** Using a dynamic imaging protocol on a full-ring CZT SPECT/CT system, we demonstrated that in ATTR-CM participants, myocardial ^99m^Tc-PYP uptake exceeded blood pool activity as early as 10 min after injection and remained stable through 2.5 h. In contrast, non–ATTR-CM participants exhibited consistently lower myocardial uptake than blood pool activity at all time points. Myocardial quantitative metrics, including SUV_mean_, SUV_max_, and %ID/mL, showed statistically significant separation between groups from as early as 10 min after injection. **IMPLICATIONS FOR PATIENT CARE:** Early quantitative ^99m^Tc-PYP SPECT/CT imaging can reliably differentiate ATTR-CM from non–ATTR-CM cases, reducing the need for a prolonged 2- to 3-h uptake period (at least for the negative cases), streamlining clinical workflows, improving participant comfort, and supporting timely disease management.
